# Cognitive flexibility supports the development of cumulative cultural learning in children

**DOI:** 10.1038/s41598-022-18231-7

**Published:** 2022-08-18

**Authors:** Sarah Davis, Bruce Rawlings, Jennifer M. Clegg, Daniel Ikejimba, Rachel E. Watson-Jones, Andrew Whiten, Cristine H. Legare

**Affiliations:** 1 School of Psychology and Neuroscience, University of St. Andrews, St. Andrews, UK; 2 Department of Psychology, Durham University, Durham, UK; 3 Department of Psychology, Texas State University, San Marcos, USA; 4 Department of Psychology, The University of Texas at Austin, Austin, USA

**Keywords:** Psychology, Human behaviour

## Abstract

The scale of cumulative cultural evolution (CCE) is a defining characteristic of humans. Despite marked scientific interest in CCE, the cognitive underpinnings supporting its development remain understudied. We examined the role cognitive flexibility plays in CCE by studying U.S. children’s (*N* = 167, 3–5-year-olds) propensity to relinquish an inefficient solution to a problem in favor of a more efficient alternative, and whether they would resist revertin*g* to earlier versions. In contrast to previous work with chimpanzees, most children who first learned to solve a puzzlebox in an inefficient way switched to an observed, more efficient alternative. However, over multiple task interactions, 85% of children who switched reverted to the inefficient method. Moreover, almost all children in a control condition (who first learned the efficient method) switched to the inefficient method. Thus, children were keen to explore an alternative solution but, like chimpanzees, are overall conservative in reverting to their first-learned one.

## Introduction

Human innovation is increasing in complexity and diversity at an unprecedented rate. The last century alone has seen dramatic technological transformations; from simple computers to smart technology and from twentieth-century steam-powered cars to self-driving automobiles. These are examples of our remarkable capacity for cumulative cultural evolution (CCE): the process of accumulating knowledge and skills that increases the complexity and/or efficiency of technology and other forms of culture over time^1–3^. CCE involves recurring cycles of invention and social transmission, in which innovations iteratively build upon previous ones, producing increasingly sophisticated cultural traits^4,5^. Although some nonhuman animals display culture—group-typical behavior that is socially transmitted—some of which may indeed be cumulative, the complexity and diversity of cumulative culture in humans is unique among other species^2,3,6^.

Behavioral flexibility is defined as “the continued interest in and acquisition of new solutions to a task, through either innovation or social learning, after already having mastered a previous solution”^7^, pg. 447. This disposition may facilitate the continual iterations involved in cumulative culture, and thus be foundational to the evolution of cultural complexity in humans, and to our ability to engage in cumulative cultural learning^3^. Conversely, while some suggest closely related species such as chimpanzees may have simpler forms of CCE^3,8^, behavioral inflexibility has been proposed as a potential explanation for relative cultural stasis in nonhuman animals. In particular, experimental studies with chimpanzees have shown they are unlikely to adopt more effective or efficient solutions to puzzles having already learned less effective or efficient ones^9–12^. Such species-specific constraints on behavioral flexibility may explain the relative dearth in cultural complexity and greater frequency of behavioral conservatism in other species^11,13^.

Behavioral conservatism and the cognitive inflexibility that underlies it is not unique to chimpanzees, however. It has been widely documented in various forms and degrees in human children^14–17^. Behavioral conservatism has been linked to cognitive control—the capacity to coordinate behavior in line with changing goals and contexts^18,19^—as well as perseveration, functional fixedness, and mental set^17,20^ (see Table 1 for related terms used by cognitive and comparative psychologists^21,22^).

**Table 1:** Common terms used in analysis of behavioral inflexibility.

Term	Definition
Cognitive flexibility	A broad term denoting the ability to adapt flexibly to a constantly changing environment^23^
Conservatism	Prior knowledge prevents or delay adoption of an alternative behavior^7^
Functional fixedness	Failing to use an object for any other purposes than its original one^17^
Mental set	The propensity to stick to the most familiar or previously successful solutions to a problem and ignore alternatives^24^
Perseveration	Failing to shift from one concept to another or to change or cease a behavior pattern once having started it^25^

Research on cognitive flexibility presents two somewhat contrasting pictures of its developmental trajectory, depending on task features. Tasks involving rule following suggest that early childhood is characterized by cognitive inflexibility, insofar as young children often perseverate on existing learned rules and fail to switch to new ones. For example, in tasks involving firstly sorting cards by one rule (e.g., color), before being asked to switch to sorting them by another rule (e.g., size), children struggle to switch to new rules up to around 5 years of age^23,26^. This cognitive inflexibility in early childhood has been explained by maturational constraints such as immature executive functions, including inhibitory control^27^, lack of relevant cultural and educational experiences^26^, and the normative adoption of observed behaviors^28,29^. Rapid improvement in cognitive flexibility that allows for rule switching over childhood may thus be critical to the development of cumulative cultural learning.

Conversely, in the absence of prior rules children appear much more flexible. Research on hypothesis searching, which involves finding the optimal causal explanation for a set of events, suggests that young children are more likely than older children, adolescents, and adults to use exploration than exploitation strategies^30^. Children often lack prior knowledge about topics they are learning about, and broad exploration strategies allow them to potentially discover novel solutions to problems. As children gain more knowledge, exploitation strategies allow children to apply prior knowledge to narrow their hypothesis space. Thus, childhood may begin with more exploration before converging on exploiting knowledge-acquisition strategies^30–32^. Constraints on cognitive control in young children may in fact allow them to *think more innovatively than adults in some contexts*. The cognitively-mature and typically adaptive reliance on prior knowledge to solve complex tasks can cause fixation on suboptimal solutions^32^.

Flexibly switching between behaviors requires a trade-off between explore and exploit strategies. Exploration strategies entail acquiring novel behaviors driven by motivation to learn about new environments. Exploit strategies, conversely, involve leaning on already acquired behaviors or exploiting known options^33^. Thus, in this framework, one way to consider cumulative culture is as an extension of behavioral optimisation, or the search for the most optimal solution within a problem space^6,34^. Why would cognitively sophisticated species such as chimpanzees exhibit behavioral conservatism given the apparent adaptive advantage of behavioral flexibility in solution optimization? The answer may lie in the potential function of behavioral conservatism. Rather than attribute the delayed maturation of cognitive control in childhood to evolutionary detritus, it may allow children to acquire the foundational skills and knowledge that adult cognition is built upon (e.g., language competence^35^). Thus, there is tension between different developmental models of children’s cognitive flexibility. Children appear more flexible in the absence of prior knowledge or explicit rules, and less so when they are present. Puzzlebox studies, which often invoke knowledge in children through prior demonstrations, support this distinction. For example, 7- to 11-year-old children who witnessed a demonstration before interacting with a multi-solution puzzlebox generated fewer novel solutions than children who did not witness a demonstration^16^.

We propose that cognitive flexibility is fundamental to CCE^12,36^, yet there has been little research into just *how* it contributes to the development of cumulative cultural learning. Our objective was to examine how cognitive flexibility supports the ontogeny of cumulative cultural learning in early childhood when children had prior knowledge. We focused on the role that the development of cognitive flexibility plays in U.S. children’s propensity to relinquish an inefficient solution to a problem, and to switch to, and maintain, a more efficient alternative.

### Behavioral switching and maintenance of new behaviors: avoiding reversion and redundancy

Cumulative culture involves the accumulation of cultural trait modifications over time, wherein successful modifications are preserved until they are again improved upon^2,37^. Successful CCE thus requires behavioral switching and maintenance of what has been adopted. The former involves relinquishing a current behavior to *switch* to an improved version, and the latter requires resisting *reverting* back to earlier versions and omitting potential *redundant* actions from previous behaviors. Together these processes allow for successful adoption and streamlined maintenance of improved cultural modifications, while preventing backward slippage^5^. Cognitive flexibility alongside other executive functions underpin skills which support innovation and social learning^38^, and thus is likely to be key to the core behaviors we argue support CCE. We next further describe each of these components, focusing on what we know of their development through childhood and how they contribute to explore versus exploit strategies, including the role of cognitive flexibility.

### Switching

CCE requires the ability to modify behavior and flexibly switch to improved alternatives^11^. Switching can be between ideas or behaviors and supports adaptation to varying environments^39^. As with all executive functions, cognitive flexibility undergoes significant development from early to middle childhood. As noted, this development improves children’s capacity to switch between tasks, rules, and cues, as well as solve problems which involve switching from previous rules or strategies. For example, the ability to switch between verbal rules for tasks such as card sorting^26,40^, touching body parts^41^, and to use semantic cues to infer meanings of novel words^26,42^, significantly improves between 3–6 years of age, and older children are better able to switch to more efficacious observed puzzlebox solutions than younger children^15^. Yet, as also noted, young western children have also been shown to outperform older children on tasks requiring flexibility in the absence of explicit prior rules. For instance, older children are more prone than younger children to functional fixedness, a bias to use objects only in the way they are traditionally used rather than thinking of new uses^17^. Younger children are also better able to learn an uncommon physical or social causal relation from evidence than are older children and adults^31^. Younger children, while having limited cognitive control, in some circumstances are thus more prone to exploration and flexibility than older children^31^. In our culturally complex worlds, this strategy may afford young children broader learning about their environments.

### Cultural trait maintenance (avoiding reversion and redundancy)

CCE also requires continued use of improved solutions over multiple task interactions without reverting back to prior, less effective behaviors^5^. To our knowledge, just one study has examined whether children revert to previously learned behaviors. Five-year-old children who initially discovered one solution to retrieve a reward from a puzzlebox before observing an alternative, equally-efficient solution, reverted to using their original solutions and also combined both solutions to innovate additional ones^43^. It remains unclear, however, when a new solution is far more efficient than the prior one, whether children will maintain the new behavior or revert to the initial one.

In addition to resisting reverting back to original behaviors, for maximal efficiency of improved ones (i.e., keeping the newly acquired behaviors as ‘clean’ as possible), individuals must also resist the tendency to perform redundant actions (for example, subcomponents of previous behaviors^38,44^). Children of all ages are known to engage in overimitation—the propensity to imitate visibly causally redundant task-related actions, such that efficiency is reduced^45,46^. Overimitation rates increase with age, however; older children deviate less from observed adult demonstrations, while younger children are more flexible in omitting them to develop their own methods^47,48^. Here, we assessed whether young children would continue to perform redundant actions after acquiring an efficient solution to a problem by observing an adult demonstrator.

### Current study

We examined cognitive flexibility from the perspective of cumulative cultural learning. We sought to reframe our understanding of the development of cumulative cultural learning from a broader perspective of why behavioral inflexibility, or limited cognitive control, in childhood may be the result of adaptive decision-making processes. We studied the factors affecting behavioral change, and conversely the factors affecting behavioral conservatism, as well as how individuals converge on solutions and use social information. We assessed U.S. 3-to-5-year old’s capacity to flexibly switch from an inefficient to an efficient solution in a puzzlebox task, and to maintain the efficient solution over subsequent trials without reverting to any components of the inefficient method. We used the same puzzlebox task previously employed by^11^ to study behavioral flexibility in chimpanzees, facilitating comparisons between the two species’ responses. Children first learned a relatively laborious and inefficient method to extract a reward from a novel puzzlebox by observing an adult, before witnessing the same experimenter perform a markedly more efficient method to gain the same reward. Children were then given multiple further opportunities to interact with the puzzlebox.

This paradigm allowed us to address three core research questions targeting each of the processes key to cumulative culture. First, we examined whether participants would show cognitive flexibility by relinquishing the learned, inefficient solution to gain a reward and switch to a more efficient one which returned the same reward. We predicted that older children would perseverate less with the inefficient solution than younger children, due to a less developed cost/benefit analysis process among younger children. This would make them more likely to change behaviour as they are less constrained by prior knowledge^30^ and thus show greater proclivity to engage in exploration for alternative solutions than older children. This is also consistent with research demonstrating greater functional fixedness in older than younger children^17^. Second, we asked whether children would resist reverting back to the inefficient solution over multiple task interactions. We predicted that because older children are more likely to evaluate the problem space due to increasing sensitivity to the risks associated with behavioural change, they would be more likely to revert to the inefficient solution due to perceived costs of change. Third, we investigated whether children would ignore redundant behaviors. We predicted that younger children would be more likely to include redundant information from inefficient solutions than older children given a greater tendency to explore the apparatus and less-developed causal reasoning. Addressing these questions allowed us to infer how the development of cognitive flexibility contributes to the ontogeny of cumulative culture. Additionally, the inclusion of a contrast condition, where children first observed the efficient version before the inefficient ones, allowed us to examine whether children would switch if they instead acquired the efficient solution first, before witnessing the inefficient one. We predicted that these children would be less likely to switch from an efficient to an inefficient solution, and more likely to revert to an efficient solution upon learning an inefficient solution.

## Results

### Inter-rater reliability

10% of videos were coded by a coder blind to the study aims. Raters showed good agreement: Kappa = 0.87.

We first quantified whether the inefficient method took longer than the efficient method by comparing the mean duration in seconds (s) to success in Phase 1 of the Inefficient first and the Efficient first conditions. Children solved the inefficient method (*M* = 33.71 s, *SD* = 12.74) significantly slower than the efficient method (*M* = 11.79 s, *SD* = 9.89, *p* < 0.001), suggesting that it was indeed much more inefficient than the efficient one.

### Did children switch solutions?

#### Inefficient first condition

**Figure 1:**
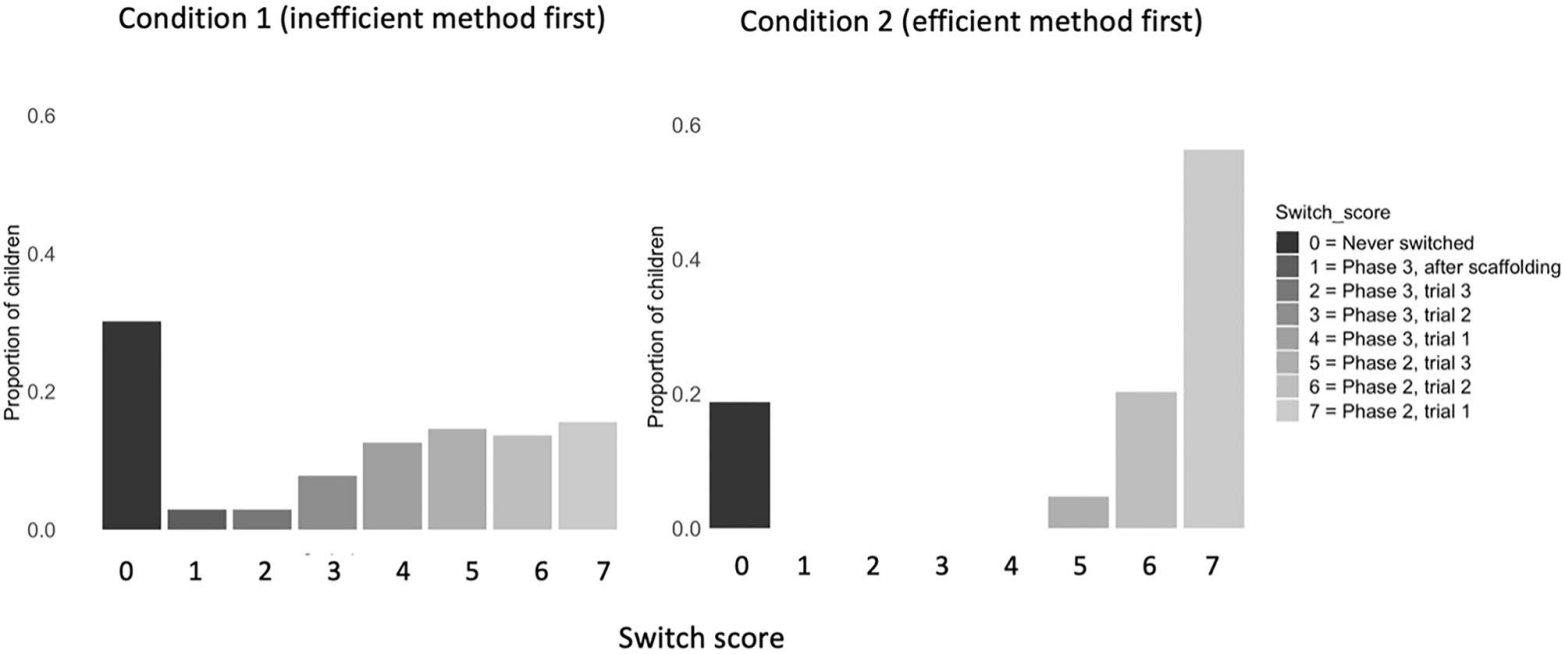
The proportion of children who switched at the different points in the experiment, by condition.

Across all phases, a total of 72 children (62%) switched from the inefficient to the efficient method once they witnessed E1 use it. When broken down into specific switch scores, not switching at all was the most frequent response (*N* = 31, 30% of participants), and switching on trial 3 of Phase 3 (when the token was in the dent), and on Phase 3 after scaffolding (i.e., the last possible opportunity) were the least frequent responses (both *N* = 3, 3% of participants); see Fig. 1 for a breakdown of the frequency and percentage of children’s switch scores. In total, of the children who did switch, 43% did so in Phase 2 (62.5% of children who switched at all) before the token was placed in the dent, and 26% of children (37.5% of those who did switch) switched in Phase 3, when the token was in the dent. The full model (AIC = 550.45) was not a significantly better fit than the null model (AIC = 497.66; deviance change = − 427.66). Neither age nor gender predicted children’s switch score in the Inefficient first condition (*p*s > 0.05). There were also no differences in the age or gender of children who switched in the first possible phase (Phase 2), suggesting 3-year-old children were as likely as 5-year-olds to be ‘early switchers’ (*p*s > 0.05). Finally, we also examined whether there was a relationship between latency to solve the Serialbox in Phase 1 and switch score. Controlling for age, children who switched earlier were quicker to solve the Serialbox (beta = − 0.01, *p* = 0.019, CI: − 0.02 to − 0.00), suggesting that those who were slower to solve it in Phase 1 tended to continue with the inefficient method for longer.

#### Efficient first condition

52 (81%) children switched from the efficient to the inefficient method once they witnessed E1 use it. The most frequent response in the Efficient first condition was to switch at the first opportunity, *N* = 32 (69% of children who switched). All children that switched did so on Phase 2, and no children switched in Phase 3—when the door was locked—suggesting that even when children had already learned an efficient method, they quickly adopted the observed inefficient method. In the Efficient first condition, males were more likely to switch than females (beta = 0.33, *p* = 0.002, CI: 0.12–0.55). Age did not predict switch score (*p* > 0.05). The proportion of children in the Inefficient and Efficient first conditions who switched method did not significantly differ (*p* > 0.05). However, the median switch score in the Inefficient first condition (*Med* = 4.00), was significantly lower than that of the Efficient first condition (*Med* = 7.00, *Z* = − 11.74, *p* < 0.001), suggesting that children who observed the efficient method first switched earlier than children observing the inefficient method first.

### Did children maintain the new solution or revert to the previous one?

#### Inefficient first condition

In total 61 children (85% of those who switched) reverted from the efficient method back to the inefficient method at least once. Children in the Inefficient first condition were generally more likely to revert later in the sequence: Phase 2 = 15% of participants who switched, Phase 3 = 13%, Phase 4 = 46%, and Phase 5 = 83% of participants who switched. The full model (AIC = 307.35) was a significantly better fit than its null equivalent (AIC = 493.47; *χ*^2^(8) = 202.12, *p* < 0.001). There was a significant positive effect of switch score (beta = 1.50, *p* = 0.001, CI: 0.58–2.41), such that children who switched later were more likely to show reversion across the task. There were significant positive main effects of phase; compared to Phase 2, children were more likely to show reversion in Phase 4 (beta = 8.19, *p* = 0.002, CI: 3.06–13.32) and Phase 5 (beta = 9.91, *p* < 0.001, CI: 3.13–13.58). There were no differences between reversion scores between phases 2 and 3, or between phases 4 and 5 (*p* > 0.05), and there was no effect of age (*p* > 0.05).

#### Efficient first condition

Across all phases, 62 children (97% of those who switched) in the Efficient first condition reverted from the inefficient to the efficient method. Children in the Efficient first condition were no more likely to revert to the original method than children in the Inefficient first condition (*p* > 0.05). As with the Inefficient first condition, reversion rates were generally higher in later phases: Phase 2 = 13% of participants, Phase 3 = 28%, Phase 4 = 53%, Phase 5 = 80%, and Phase 6 (transfer of knowledge) = 45% of participants (see Table 2 for comparisons of reversion across conditions and phases). Neither age nor gender predicted reversion score (*p*s < 0.5) in the Efficient first condition.

**Table 2:** The proportion of children reverting to their originally learned solution by condition and phase.

Phase	Inefficient first condition (%)	Efficient first condition (%)
Phase 2	15	13
Phase 3	13	28
Phase 4	46	53
Phase 5	83	80
Phase 6	–	45

### Did children perform redundant actions?

#### Inefficient first condition

Children’s redundancy scores were highest in Phase 3, when the inefficient method was blocked, and lowest in Phase 5; Phase 1: *M* = 0.26, *SD* = 0.70, Phase 2: *M* = 1.21, *SD* = 1.76, Phase 3: *M* = 2.11, *SD* = 1.85, Phase 4: *M* = 0.31, *SD* = 0.66, Phase 5: *M* = 0.30, *SD* = 0.75. The full model (AIC = 1150.9) was significantly a better fit than its null equivalent (AIC = 1817.5; *χ*^2^(10) = 686.59, *p* < 0.001). There was a significant negative effect of age (beta = − 0.27 *p* = 0.001, CI: − 0.48 to − 0.065); younger children showed higher redundancy scores than older children. There were also main effects of phase; compared to Phase 1, children showed higher redundancy scores in Phase 2 (beta = 1.27 *p* = 0.001, CI: 0.49–2.05) and Phase 3 (beta = 2.20 *p* < 0.001, CI: 1.46–2.93). There were no differences between Phase 2 and Phases 4 or 5 and there were no significant main effects of switch score or switch score by phase interactions (all *p*s > 0.05).

#### Efficient first condition

The highest average redundancy score was in Phase 1 (when children were learning the efficient method) and the lowest was in Phase 3 (door locked); Phase 1: *M* = 2.16, *SD* = 2.33, Phase 2: *M* = 1.56, *SD* = 1.77, Phase 3: *M* = 0.30, *SD* = 0.75, Phase 4: *M* = 0.72, *SD* = 0.95, Phase 5: *M* = 0.41, *SD* = 0.79, Phase 6: *M* = 0.65, *SD* = 0.92. Age negatively predicted redundancy score (beta = − 0.038, *p* < 0.001, CI: − 0.59 to − 0.19), such that younger children produced more redundant actions than older children. Switch score positively predicted redundancy score, such that children who switched to the inefficient method earlier produced more redundant actions than those who switched later (beta = 0.070, *p* = 0.049, CI: 0.00–0.13). Across phases, there were no differences in median reversion scores for children in Inefficient first condition (*Med* = 0.00) and Efficient first condition (*Med* = 0.00, *p* > 0.05).

## Discussion

Our core objective was to examine how cognitive flexibility supports the ontogeny of cumulative culture. We assessed two processes key to CCE in US 3–5-year-old children: (1) switching to a witnessed, more efficient solution to a problem and (2) maintaining this through resisting reversion to the earlier solution and omitting potential redundancies consequent on the change.

### Did children switch to alternative solutions?

Over multiple task interactions, around two thirds of children in the Inefficient first condition switched to the efficient method. Contrary to our hypothesis, there were no age differences in the propensity to switch solutions. Thus, even at 3 years, children can flexibly relinquish an inefficient yet reliable behavior to adopt a more efficient one they witness another perform, and which they had not discovered by themselves. The fact that of those that did switch, most did so in Phase 2 rather than Phase 3 (i.e., in the first available phase) suggests that children quickly assessed the efficiency of the two witnessed solutions. The findings contrast with those from cognitive flexibility studies in which when young children were provided with instructions to switch behaviors but no actual demonstrations, they failed to switch^23,26^. Given that children showed a strong proclivity to return to their original method, it is possible that here, the 3 and 5 year old children were using exploratory strategies similarly, or that the children, irrespective of age, are particularly faithful to initially-witnessed adult demonstrations despite the availability of alternative (and more efficacious) solutions^15,16^. It is also possible that our age range was too narrow to capture quantitative developmental shifts in the switching strategies assessed in this study. Future work could assess children’s proclivity to switch and/or revert to solutions across the entire childhood period, and whether the propensity to switch methods is impacted by the age of the model (i.e., adult model vs child model).

We also found that children who solved the Serialbox quicker in Phase 1 of the Inefficient first condition tended to switch earlier, while those who were initially slower to solve it were those who switched later in the task. Previous work has shown that cognitive flexibility in card sorting and word meaning tasks is associated with response speed in marking boxes^23^. Our findings, in the puzzlebox domain, are in line with these and may thus suggest that those who solved it faster were quicker to grasp the affordances of the Serialbox (and thus that the efficient method was a more efficient way of solving the task).

More striking was that all children in the Efficient first condition switched to the inefficient method, and most did so at the first opportunity. This suggests that children’s general strategy may have been to acquire new information about the Serialbox by exploring different solutions. Previous work has shown that young children can be more flexible and exploratory learners than older children, perhaps because they have less executive control^31^. Early capacities for flexibility and exploration may be an adaptive mechanism allowing young children to acquire a wide range of cognitive skills, including promoting innovation^30,31^. Further, children may be more inclined to switch on practical tasks such as gaining rewards from our Serialbox compared to other, more abstract measures of cognitive flexibility that typically involve the application of rules such as sorting cards by size.

Conversely, in experiments with the same puzzlebox^11^, just 18% of chimpanzees, compared ﻿to 56% of children here, switched to the efficient method prior to the token being put into the dent (phase 3 in this study). Even after the token was in the dent, only 45% of chimpanzees switched compared to 62% of children here. This contrast between child and chimpanzee responses may be one factor contributing to the enormous differences in cultural technology between the two species. Cumulative culture requires adopting improved behaviors, and young children’s greater motivation to rapidly switch to alternative, more efficient behaviors suggests that the cognitive processes supporting CCE are early-developing. Such findings may also provide insights into the evolutionary trajectory of cognitive flexibility. For example, the gradual transition from stone tools to modern tools presumably required flexibility to modify and improve existing technology. Indeed, recent research showing variation in stone tool technology in *H. erectus* of Gona, Ethiopia, suggests they were capable of impressive flexibility^49^.

It is, however, important to note that in^11^, chimpanzees performed the inefficient behavior a minimum of 20 times before observing the efficient method, while in this study children could perform it only twice before they observed the alternative demonstration. Increased familiarity with task functions and success are known to enhance conservatism in both species^15,17,50,51^. Future work could examine how varying levels of prior exposure to, and habitual use of, less efficient solutions impact children’s propensity to switch to improved ones﻿.

### Maintaining a new, improved, behavior through avoiding reversion to the previous approach and omitting redundant actions

We also investigated children’s proclivity to maintain the new, more efficient behavior by avoiding reversion to the inefficient one and/or ignoring redundant actions in the transition. Most children in both conditions who did switch to the alternative method reverted to their initially acquired solution (85% and 97% for the Inefficient and Efficient first conditions, respectively). By contrast, just 20% of chimpanzees reverted to the inefficient Serialbox method having learned the efficient one^11^ —though the chimpanzees were tested in groups, and the lack of reversion may have reflected using the most frequent strategy^44^. Reversion behaviors was found in some previous puzzlebox work with similarly aged children. After individually learning a new (similarly efficient) puzzlebox solution, 4- to 6-year-old children reverted to their originally acquired socially learned solutions, but also added components of the new behavior, which led to innovations^51^. Here, when the alternative method was socially demonstrated, and when given multiple trials, children tended to return to their original solution. Children are strongly influenced by, and remain faithful, to initially socially-acquired solutions in such technical contexts^15,16,51,52^. Plausibly, children were keen to explore the observed alternative solution but were overall conservative in reverting to their first-learned method across task interactions. This may explain why almost all children in the Efficient first condition reverted to the inefficient solution.

Reverting to an inefficient method appears an irrational strategy—in the same way that overimitation (copying causally irrelevant actions), a behaviour not found in chimpanzees^53^ has been often characterised as such. Yet, overimitation appears to serve at least two adaptive functions. Firstly, it helps children to rapidly acquire information regarding object affordances from our complex causally opaque, tool-rich environments. Second, in a world surrounded by unintuitive customs, norms and rituals, copying adults with extreme fidelity also allows children to assimilate to their social environments^45,46^. Children’s proclivity to revert to the inefficient method here thus mirrors overimitation insofar as their copying overrides using the more efficient solution from a causal perspective.

These findings have important implications for our understanding of both the ontogeny of cumulative cultural evolution and comparative cultural cognition, as in the contrasts with chimpanzee behavior outlined above. CCE requires sustained use of improved behaviors such that they can establish within populations. Our findings that over multiple attempts, instead of continuing with a more efficient behavior, young children are inclined to revert to a less efficient one may suggest that conservatism is still present in young children when task parameters allow (i.e., multiple opportunities for task interaction). Future work could investigate whether this tendency to revert continues into middle and late childhood, to assess whether the development of cognitive flexibility, and other executive functions, impacts children’s proclivity to continue to use more efficient behaviors rather than revert to less efficient ones. Likewise, population size, structure and connectivity influence the accumulation of innovations^54^, and future work should also assess whether children show reversion or maintain improved solutions when solving tasks in group settings.

Overall, children performed some redundant actions (e.g., lifting lids when not necessary), though in most phases rates were low. Keeping the newly acquired behaviors as ‘clean’ as possible requires ignoring redundant actions, and children’s tendency to resist using them is in line with previous research showing that over multiple interactions and experimental generations, children progressively develop streamlined methods for solving technical tasks^55^. Younger children performed more redundant actions than older children, potentially reflective of a ‘division of labor’ over developmental time^30,31^. These findings converge with previous work suggesting younger children tend to use exploratory strategies for problem solving, in which they readily adopt new hypotheses regarding causal relations from observed actions^30,31^. With age, greater accumulation of knowledge and executive control, children become better equipped to exploit optimal social information and can rely on their existing causal knowledge^30,31^. These developmental changes may explain how innovations are adopted and transmitted. Young children find goal-directed innovation, a skill requiring multiple executive processes, very difficult^56–58^, yet are remarkably adept at imitating observed innovations^56^.

### Did switching behavior impact reversion and redundant actions?

Compared to children who took more trials to switch, those who switched earlier showed fewer subsequent reversions to the inefficient method and used fewer redundant actions—both of which are important steps for establishing solid cumulative cultural improvement. These findings provide new evidence that children who more readily acquire improved solutions are those who also tend to promote the maintenance of improved cultural traits. This may have important implications for our understanding of the ontogeny of cumulative culture, in particular whether certain individuals are key to its expression^59–62^. This finding also highlights the importance of using study paradigms affording assessment of the different components of CCE, such as providing extensive task interaction opportunities. Further work could attempt to document whether there are certain characteristics which predict whether children are likely to be early switchers, as has been done with UK children’s propensity to imitate or deviate from adult demonstrations^16^.

## Conclusion

Cumulative cultural evolution is contingent on the capacity to flexibly switch to, and maintain, improved behaviors. Our findings shed light on the role cognitive flexibility plays in cumulative culture by showing that, in contrast to chimpanzees, young children can readily relinquish inefficient task solutions to switch to more efficient ones. Yet children also showed a strong proclivity to return to initially learned solutions, suggesting a robust tendency to rely on initially learned solutions from adults—even if they are less efficient. In showing that children quicker to switch solutions were also those that were more likely to maintain them over multiple task interactions, our findings also provide new insights into which individuals are key to the uptake and preservation of improved cultural traits.

## Methods

### Participants

194 children aged 3–5 years participated. Twenty-seven participants were excluded due to experimental error, leaving a total of 167 children (*M* age = 4.50 years, *SD* = 0.83; 85 females). Data were collected at the Thinkery Children’s Science Museum, Austin, Texas, USA, in a room not accessible to the general public. The five largest ethnic groups in the surrounding county (Travis County) are White (48.7%), Hispanic (32.16%), Black (7.92%), and Asian (6.81%).

### Ethical note

This study was approved by the Department of Psychology, University of Texas at Austin’s Institutional Review Board (IRB) committee (IRB study number 2010-06-0059). Parental/guardian informed consent was obtained at the Children’s Science Museum prior to testing. The experimental procedures were performed in accordance with the University of Texas at Austin’s guidelines and regulations.

### Experimental puzzle box task: Serialbox

**Figure 2:**
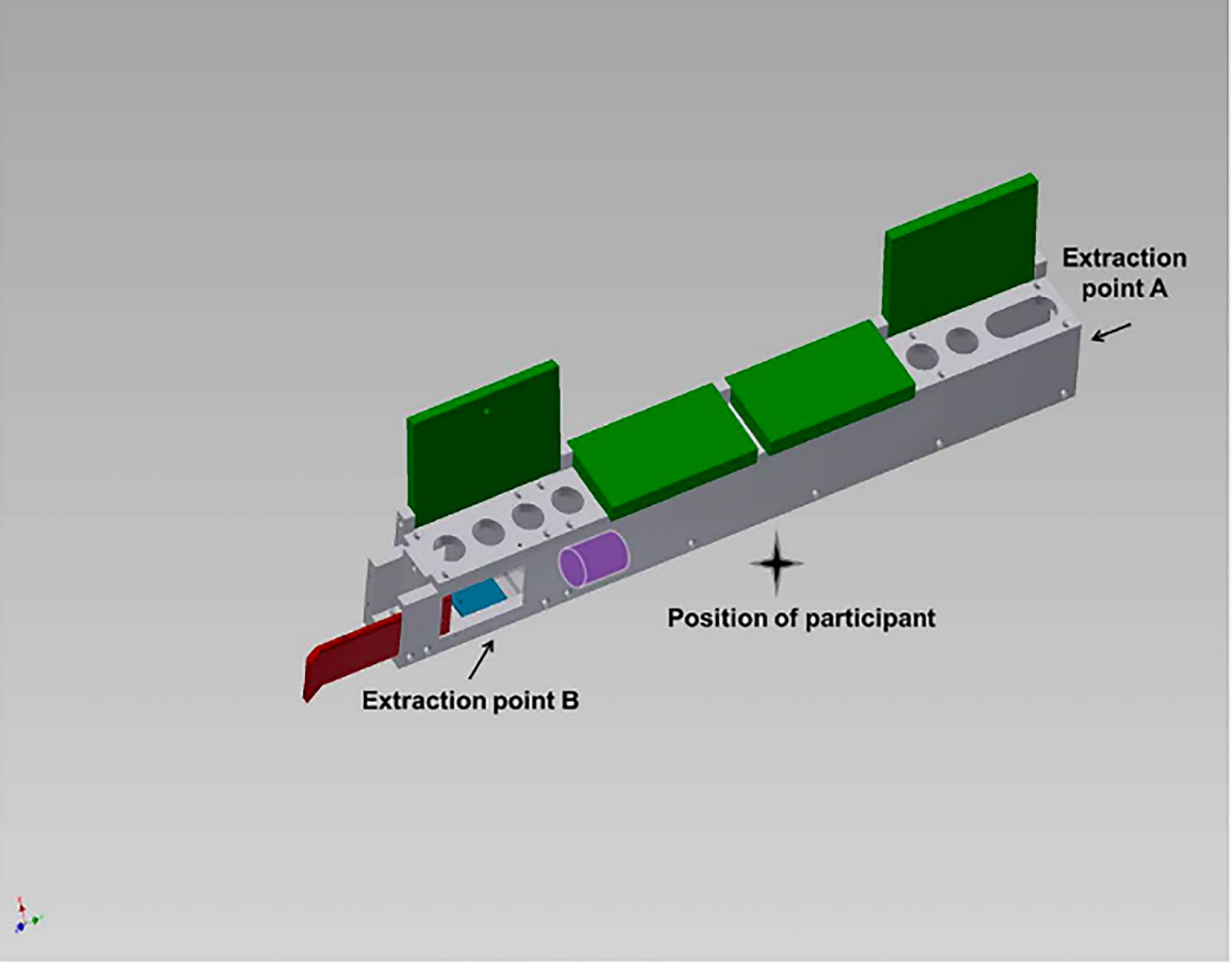
The Serialbox. Figure taken from Ref.^11^.

The Serialbox (Fig. 2) was a transparent polycarbonate apparatus (61 cm × 46 cm × 7 cm) with four compartments. This version was a slightly smaller, ‘child-sized’ version of the Serialbox used with chimpanzees by^11^. Each of the four compartments contained a hinged lid which, when lifted, exposed four finger holes per compartment. These 2.5 cm × 2.5 cm finger holes allowed access to a white cylinder token, which was initially located in the furthest-left compartment, under the third and fourth finger holes (left-most compartment*,* Fig. 2). There were two possible methods to retrieve the token: an *inefficient* and an *efficient* method. The *inefficient* method involved moving the token from one end of the apparatus to the other by lifting each of the four compartment lids and using the finger holes to nudge the token along. This method moved the token from the left-most compartment to an access point at the far-right compartment (*Extraction point A*, Fig. 2). The *efficient* method involved pulling open a door (*Extraction point B*, Fig. 2) on the far-left compartment, accessible from the outside of the Serialbox on the participants’ side, to access the reward. Additionally, a dent in the floor on the right side of the far-left-most compartment (in blue in Fig. 2) made the token very difficult to extract when initially placed there.

### Procedure

**Figure 3:**
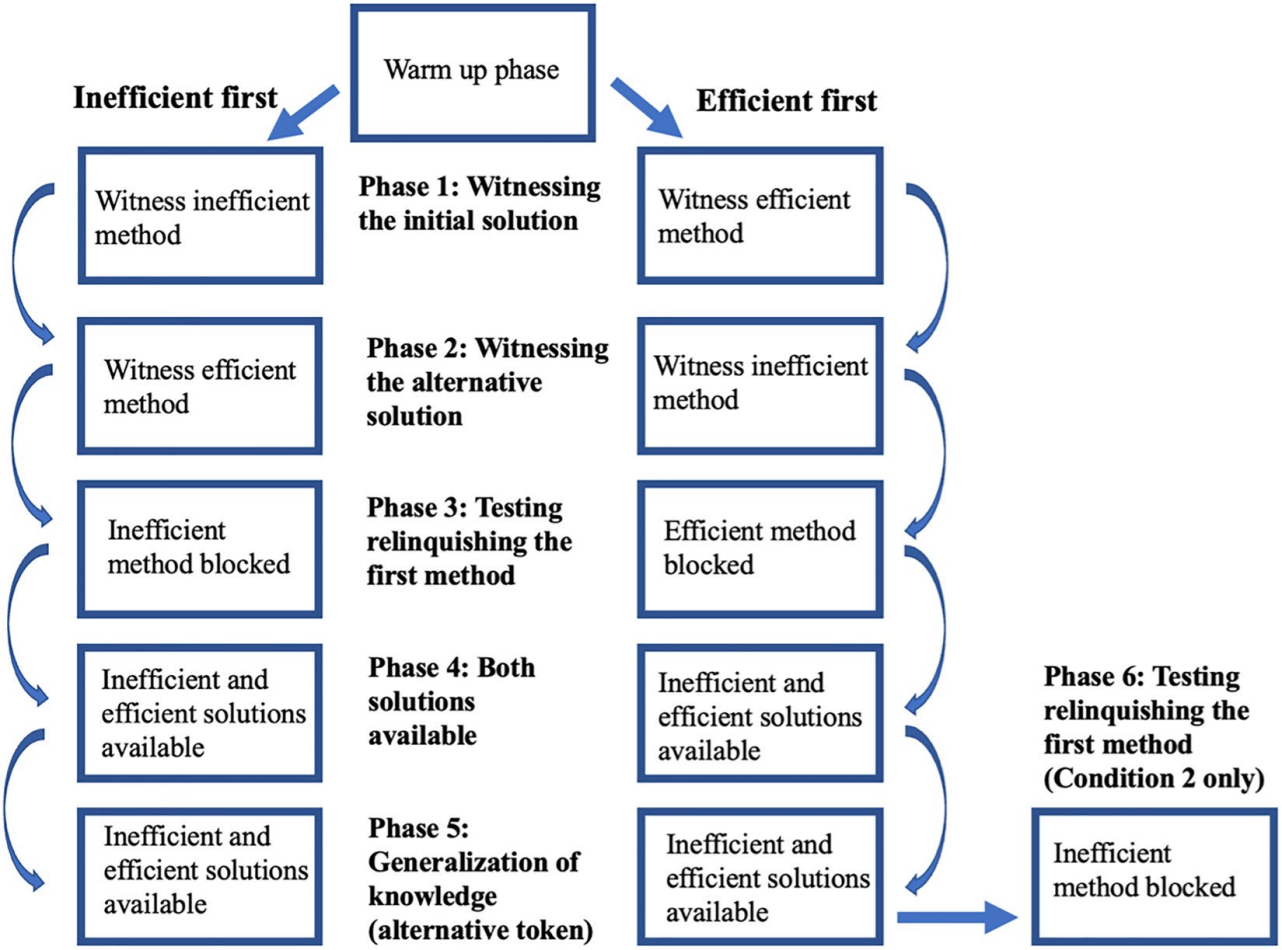
Breakdown of the experimental design.

The experiment began with a warm-up exercise between experimenter and participant which involved reading a story to build rapport. Children were then presented with the Serialbox and testing began. The experiment consisted of either five or six sequential phases, with children participating in one of two conditions depending on whether the inefficient or efficient method was presented first: Inefficient first condition: inefficient to efficient, *N* = 103 (51 females), *M* age = 4.48, *SD* = 0.81, Efficient first condition: efficient to inefficient, *N* = 64 (34 females), *M* age = 4.52, *SD* = 0.85. Inefficient first condition data were collected before Efficient first condition data were collected. For a breakdown of the experimental design, see Fig. 3. All testing sessions were video recorded with a discretely placed video camera in the corner of the testing room and two experimenters were present during testing sessions. In all cases, between trials, the token was replaced in its starting position in the Serialbox out of sight of participants. In addition, participants were rewarded after the experiment with a sticker regardless of method used or success. To maintain consistency in experimenter interaction across participants, the dialogue was restricted to the script as outlined below, and the experimenter did not assist the participant during task interaction.

### Phase 1: Witnessing and learning the initial solution

Children first learned one solution to the Serialbox, which was pre-loaded with a token at the left-most compartment (Fig. 2). Experimenter 1 (E1) pointed to the token and said: “*Do you see that white thing in the box? Every time one of us gets it out, you get a prize. Okay? It’s my turn.*” Experimenter 1 then demonstrated either the inefficient method (in the Inefficient first condition) or the efficient method (Efficient first condition), before saying “*I got it out*”. Experimenter 1 then took the participant to a separate table to choose a sticker reward (for distraction) while Experimenter 2 reset the apparatus. Experimenter 1 then said: “*It’s your turn*”. If the participant successfully retrieved the token, they were given the opportunity to have one additional attempt before moving to Phase 2. If they failed to attempt the inefficient method (Inefficient first condition) or efficient method (Efficient first condition) within 1 min, they were provided with up to three further demonstrations and attempts before moving to Phase 2 (all participants progressed).

### Phase 2: Witnessing the alternative solution

Children witnessed the alternative solution in Phase 2. At the start of this phase, the token was already repositioned at the left-most compartment. The procedure was identical to that used in Phase 1 except that Experimenter 1 demonstrated the method that the child had not seen before (either the efficient method in the Inefficient first condition or the inefficient method in the Efficient first condition). After the initial demonstration, participants who switched and used the new method were then given one more demonstration and attempt before moving to phase three (regardless of the method they used in their second attempt). Those who used the alternative method from the demonstration (i.e., the method demonstrated in Phase 1 or the inefficient method in Inefficient first condition or the efficient method in Efficient first condition) were given up to two more demonstrations and attempts to use the efficient (Inefficient first condition) or inefficient (Efficient first condition) method. Following this, all participants, regardless of the extraction method they were using, moved to Phase 3.

### Phase 3: Testing for relinquishing a highly inefficient or efficient method

To examine whether when faced with a highly inefficient solution children would switch to an efficient one (and vice versa), in Phase 3 the first method children acquired was made much more difficult. For the Inefficient first condition, the token was placed inside the indent in the left-most compartment (close to Extraction point B, see Fig. 2). The indent made it much more difficult to extract the reward using the inefficient method (and conversely, comparatively far more effective to use the efficient method^11^. No demonstrations were initially provided. If the participant used the efficient method to extract the token, they were offered one more attempt before proceeding to Phase 4 (regardless of which method they used on their second attempt). If participants failed to extract the reward after 1 min, or used the inefficient method on their first attempt, Experimenter 1 demonstrated the efficient method before allowing the participant up to two more attempts to retrieve the token (using either method). If after three trials participants continued to not use the efficient method, Experimenter 1 held the door at Extraction point B partially open to scaffold the efficient method and gave the participant one more attempt. For coding purposes, this step is referred to as “scaffolding”. After this, regardless of method used, participants moved on to Phase 4.

For the Efficient first condition, the method and demonstrations were identical to Phase 2-Conditon 2, except that the door was locked shut (such that only the inefficient method was a viable strategy). If the participant pushed the token into the indent, after five seconds, Experimenter 1 replaced the token close to extraction point A (everything else was left the same such that any open compartment lids were left open). If after two attempts the child did not retrieve the token, the experimenter scaffolded their efforts by placing the token at the inefficient extraction point. This was considered the “scaffolding” step for coding purposes.

### Phase 4: Inefficient and efficient solutions available

Phase 4 examined whether children would continue to use the alternative solution or to revert back to their originally-acquired one. Phase 4 was almost identical for both conditions. The token was repositioned in the left-most compartment. To emphasize that the token was no longer in the indent (Inefficient first condition), Experimenter 1 said “*I need to check something*” before moving the token slightly left and right and repositioning it in the left-most compartment. To demonstrate that the door was unlocked (Efficient first condition), Experimenter 1 said “*Let me check something* and opened and closed the door. Experimenter 1 then said: “*It’s your turn*”, and participants were given one attempt to retrieve the reward with no demonstration. Regardless of method used or success, participants then proceeded to Phase 5.

### Phase 5: Generalization of knowledge

Phase 5 assessed whether children would generalize their behavior to a new token. Phase 5 was again identical for both conditions. An alternative token (a small cork token) was repositioned in the left-most compartment. Experimenter 1 then said, “*It’s your turn*” and participants were given one attempt to retrieve the reward using any method, with no demonstration.

### Phase 6 (Efficient first condition only): Testing relinquishing of the second method

To measure how children in the Efficient first condition would behave when the inefficient method was made highly difficult, the original token was replaced inside the indent of the left-most compartment, such that it replicated Inefficient first condition Phase 3. Experimenter 1 then said, “*It’s your turn*” and participants were given one attempt to retrieve the reward using any method, with no demonstration.

### Coding

All video data were coded by research assistants to capture three behaviors key to CCE: switching, reversion and redundancy. These were defined and coded as follows:

#### Switching

Switching denotes switching from the inefficient to the efficient method (Inefficient first condition) or from the efficient to the inefficient method (Efficient first condition). Switching score was calculated only for the first three phases. All participants were given a score between 0–7 denoting their switching score. These were scored such that higher scores represent earlier switching while lower scores represent later switching:

- 7 = switching on Phase 2, trial 1
- 6 = switching on Phase 2, trial 2
- 5 = switching on Phase 2, trial 3
- 4 = switching on Phase 3, trial 1
- 3 = switching on Phase 3, trial 2
- 2 = switching on Phase 3, trial 3
- 1 = switching on Phase 3 after scaffolding
- 0 = never switched

#### Reversion

Reversion scores were calculated for phases 2, 3, 4, 5 and 6 (Phase 6 was for the Efficient first condition only) and reflected whether participants reverted back to their originally used method, having already performed the alternative method. In the Inefficient first condition this indicated participants reverting to the inefficient method after having performed the efficient method. In the Efficient first condition this indicated participants reverting to the efficient method after having performed the inefficient method. For each phase, participants were given a score of 0 or 1 to reflect whether they showed reversion at any point within the phase.

#### Redundancy

Redundancy scores reflect participants performing redundant behaviors. For all phases, two types of redundant behaviors were calculated and summed: lid and door redundancy. For lid redundancy participants were scored a value of 0 for no redundant lid opening, a value of 1 for opening and closure of relevant lids, value of 2 for completely unnecessary opening of lids. For door redundancy, participants were scored 0 if the door was never opened, 1 if the door was opened then shut/reopened and eventually used in efficient extraction, or a value of 2 if the door was opened but never used. Thus, the redundancy score was, for each phase, the sum of the lid and door redundancy scores.

### Statistical analyses

Experimental condition data were analyzed using either multiple regressions or generalized mixed effects models. Switch scores were analyzed using a multiple regression with a Poisson fit, with age and gender entered as fixed factors. Reversion and redundancy data were analyzed using generalized mixed effects models, with either ordinary least squares (OLS), logistic, or Poisson fits, and with fixed effects of age, gender, phase, switch scores and phase by switch score interactions, and participant ID included as a random effect. In all models, the earliest phase in which the coded data was collected (either phase one or two) was used as the reference category for main effects analyses and phase by switch score interactions. For the control group data (Efficient first condition), descriptive statistics are presented along with the corresponding multiple regression results in which age and gender were entered as predictor variables. To assess differences between the two conditions, median or frequency scores were compared using nonparametric tests.

## Data Availability

Data is available on the repository Dryad (10.5061/dryad.00000005r).
